# Kommunale Alkoholprävention in Deutschland: Strukturen, Strategien und Herausforderungen

**DOI:** 10.1007/s00103-021-03334-9

**Published:** 2021-05-22

**Authors:** Thomas Praßer, Hans-Jürgen Hallmann, Michaela Goecke

**Affiliations:** 1 Referat 1–13, Prävention des Substanzmissbrauchs, Suchtprävention, Bundeszentrale für gesundheitliche Aufklärung (BZgA), Maarweg 149–161, 50825 Köln, Deutschland; 2 Ginko Stiftung für Prävention, Mülheim an der Ruhr, Deutschland

**Keywords:** Kommune, Alkoholmissbrauch, Prävention, Strategie, Programme, Municipality, Alcohol abuse, Prevention, Strategy, Programs

## Abstract

Die kommunale Alkoholprävention ist ein wichtiges und vielschichtiges Arbeitsfeld der Suchtprävention. Eingebettet in das Subsidiaritätsprinzip wird sie durch verschiedene Vorgaben und Rahmenbedingungen geprägt: etwa durch die Verabschiedung des Präventionsgesetzes (PrävG) 2015 und die Etablierung der Nationalen Strategie zur Drogen- und Suchtpolitik 2012 durch den Bund. Die detaillierte Gestaltung alkoholpräventiven Handelns obliegt allerdings Ländern und Kommunen und ist häufig an Rahmenbedingungen und Herausforderungen vor Ort geknüpft.

In diesem Beitrag werden die unterschiedlichen Strategien und Organisationsstrukturen kommunaler Alkoholprävention einführend dargestellt und diskutiert. Probleme, mit denen Kommunen bei der Umsetzung alkoholpräventiver Interventionen konfrontiert sind, werden ebenso betrachtet, wie die Möglichkeiten und Bedingungen, die Gemeinden für die Etablierung einer qualitätsgesicherten Alkoholpolitik in den Blick nehmen müssen. Außerdem werden vielversprechende Ansätze aus lokal agierenden Modellprojekten dargestellt.

Einigkeit in Politik, Forschung und Praxis besteht darin, dass Alkoholprävention am besten kommunal betrieben wird und dass die Bedeutung der Kommunen bei der Umsetzung alkoholpräventiver Interventionen zugenommen hat. Daraus ergibt sich die Notwendigkeit einer konsequenten Stärkung dieses Handlungsfelds. Diese kann durch eine verbesserte Qualifizierung von Fachkräften und einen stetigen Wissenschaft-Praxis-Transfer aktueller Forschungsergebnisse und Best Practice realisiert werden. Des Weiteren sollten Praktikerinnen und Praktiker Beratung und Unterstützung (etwa bei der Identifizierung individueller Bedarfe) durch koordinierende Stellen erhalten. Lokale Initiativen sollten einen besseren Zugang zu wirksamkeitsgeprüften Interventionen erhalten und deren nachhaltige Verankerung und Evaluation im kommunalen Setting sind anzustreben.

## Abstract (en)

Alcohol prevention in municipalities is an important and complex aspect of drug prevention in Germany. Based on the principle of subsidiarity, prevention measures are characterised by different legal requirements and prevailing conditions. On the one hand, the federal government set a legal framework with the law of prevention (PrävG) in 2015 and established the national strategy of drug policy and prevention in 2012. On the other hand, municipalities are responsible for the detailed design of local drug policy and interventions together with federal states. Furthermore, preventive measures need to fit local challenges and conditions.

Hence, we discuss different strategies of alcohol prevention in municipalities and explain how these strategies fit in administrative frameworks. We also look critically at specific local situations and challenges but also at conditions and possibilities to establish a quality-assured local alcohol policy. Promising approaches from pilot projects are contributed to the discussion.

Political decisionmakers, scientists and skilled workers accord that alcohol prevention needs to be implemented in municipal settings and that it is a field of growing importance. We conclude that there is a need to improve local prevention measures by creating better access to qualification programs for skilled workers in combination with a constant transfer between science and pratice of current research results and best practices by providing consultation and support from coordinating institutions for local interventions, especially for the identification of challenges and conditions. Local initiatives should receive better access to interventions that are proven to be effective, and these interventions should be sustainable anchored and evaluated in their municipal settings.

## Einleitung

Die Alkoholprävention ist ein Handlungsfeld der Suchtprävention bzw. der Gesundheitsförderung. Eingebettet in das Subsidiaritätsprinzip bewegt sich die Alkoholprävention innerhalb eines komplexen Handlungsrahmens, der einerseits auf einer Arbeitsteilung zwischen Bund, Ländern und Kommunen basiert, andererseits durch die Zuständigkeiten der Kranken- bzw. Sozialversicherungen sowie die Zusammenarbeit mit Leistungserbringern und nichtstaatlichen Akteuren geprägt ist [1].

Ziel dieses Artikels ist einführend über die Struktur der Sucht- und Alkoholprävention in Deutschland und über die Kommune als zentrales Setting der Alkoholprävention zu informieren und gegenwärtige Herausforderungen, Strategien und Ansätze zu diskutieren. Abschließend soll dargestellt werden, wie gelingende Alkoholprävention in Kommunen verankert werden kann.

## Suchtprävention in Deutschland

**Figure 1:**
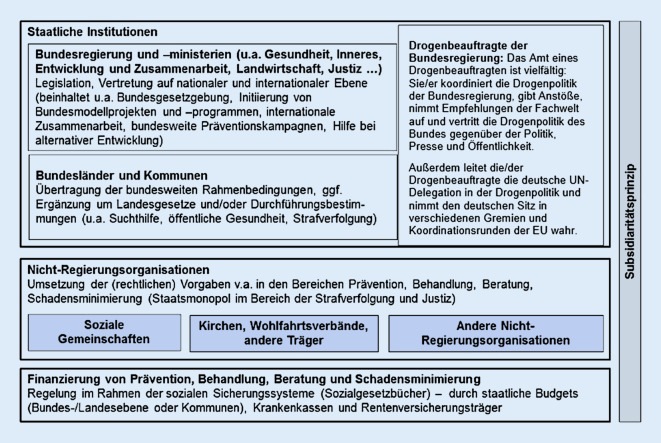

Innerhalb der Zuständigkeiten für Suchtprävention (Abb. 1) obliegt dem Bund die Gesetzgebungskompetenz. Auf dieser Grundlage setzt die Bundesregierung den rechtlichen Rahmen für die Drogen- und Suchtpolitik und gibt deren Standards vor. Dabei verfolgt sie die Strategie einer ganzheitlichen Drogen- und Suchtpolitik, die auf 4 Säulen fußt: Neben der Behandlung von Suchtkrankheiten, der Überlebenshilfe für Betroffene und dem repressiven Einschreiten gegen suchtkriminelles Verhalten ist die Suchtprävention eine dieser 4 Säulen [2].

Die Ausführung der Bundesgesetze und die Gestaltung der gesetzten Rahmenbedingungen sind im Wesentlichen die Aufgaben von Bundesländern und Kommunen. Der Bund übernimmt hingegen koordinierende, übergreifende und strukturentwickelnde Aufgaben mit seinen Institutionen Bundesministerium für Gesundheit, Drogenbeauftragte der Bundesregierung, Bundeszentrale für gesundheitliche Aufklärung (BZgA). Als Beispiel ist hier der „BZgA-Länder-Kooperationskreis für Suchtprävention“ zu nennen, durch den die Abstimmung von bundes- und landesweiten suchtpräventiven Maßnahmen ermöglicht wird, indem sich Akteure vernetzen und die Qualitätssicherung in der Suchtprävention vorangetrieben wird.

Ländern und Kommunen sind gemäß dem Subsidiaritätsprinzip wesentliche gestalterische Kompetenzen eingeräumt, die ihnen die Möglichkeit bieten, eigene Strategien zur Sucht- bzw. Alkoholprävention zu entwickeln und umzusetzen. Unterstützt werden sie dabei durch Kranken- und Sozialversicherungen sowie nichtstaatliche Organisationen, die im Auftrag oder im Eigeninteresse Suchtprävention betreiben. Oft sind die Herangehensweisen dieser Akteure an die jeweiligen Gegebenheiten und Herausforderungen vor Ort angepasst. Vor diesem Hintergrund ist eine übergeordnete Steuerung und Koordinierung nur begrenzt möglich – als ein Instrument bietet sich die Gestaltung von Förderrichtlinien an [3]. Wenn man die Sucht- bzw. Alkoholpräventionslandschaft betrachtet, ergibt sich ein diversifiziertes Bild aus differenzierten Organisations- und Finanzierungsstrukturen, Strategien, Kompetenzen und Akteursgruppen.

## Das Präventionsgesetz und die Ziele der Alkoholprävention

**Figure 2:**
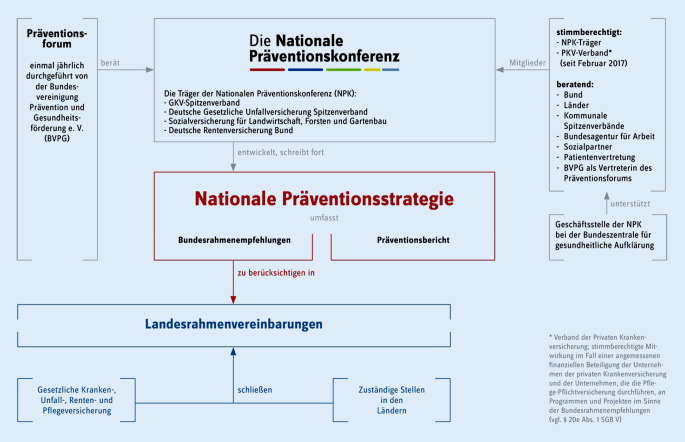

Mit dem im Jahr 2015 verabschiedeten Präventionsgesetz (PrävG) wurde u. a. das Ziel gesetzt, Gesundheitsförderung und Prävention mit einer Strategie auszustatten. Diese „Nationale Präventionsstrategie (§ 20d SGB V)“ wird durch die Nationale Präventionskonferenz (NPK) entwickelt (Abb. 2) und umfasst die Vereinbarung bundeseinheitlicher und trägerübergreifender Rahmenempfehlungen zur Gesundheitsförderung und Prävention (Bundesrahmenempfehlungen) sowie die vierjährliche Publikation des Präventionsberichts, der die zurückliegende Entwicklung beschreibt und evaluiert. Mit der Strategie sollen Gesundheitsförderung und Prävention in Lebenswelten gestärkt und nationale Gesundheitsziele erreicht werden.

Verantwortliche in Lebenswelten, z. B. Kommunalverwaltung, Fach- und Lehrkräfte, freie Träger, lokale Initiativen usw. werden durch § 20a SGB V (PrävG) mit dem Auftrag einer „Gesundheitsförderung und Prävention in Lebenswelten“ [6] ausgestattet. In der Umsetzung sollen sie dabei mit den Trägern der NPK auf kommunaler Ebene zusammenarbeiten. Eine direkte Aufgabenübertragung an die Kommunen ist im Präventionsgesetz nicht vorgesehen. Trotzdem kommt ihnen „eine Schlüsselrolle bei der Definition und Umsetzung präventiver und gesundheitsförderlicher Politiken zu“ [7].

Für die Alkoholprävention ist das PrävG von Bedeutung, da darin die Erreichung der nationalen Gesundheitsziele verankert ist. Somit ist das Ziel „Alkoholkonsum reduzieren“ [8] für die Alkoholprävention leitend. Dieses Ziel steht analog und Bezug nehmend auf die internationalen Gesundheitsziele der Weltgesundheitsorganisation (WHO) und der Europäischen Union (EU): „Reduce harmful use of alcohol“ [9].

Die Alkoholprävention verfolgt außerdem das Anliegen, die Gesundheit jedes Einzelnen, also der Allgemeinbevölkerung, zu fördern, „Abhängigkeiten vorzubeugen und den problematischen gesundheitsgefährdenden Konsum zu verringern“ [2]. Dabei werden die Zielgruppen systematisch in ihren Lebenswelten erreicht und es wird angestrebt, eine gesundheitsförderliche Veränderung von Wissen, Einstellungen und Verhaltensweisen zu bewirken sowie den Erwerb von Lebens- und Risikokompetenzen zu fördern [10]. Kinder und Jugendliche sind dabei eine besonders wichtige Zielgruppe: Denn Alkoholkonsum ist gerade im frühen Jugendalter besonders schädlich für den Körper – insbesondere das Rauschtrinken^1^. Dieses sollte im Jugendalter möglichst unterbunden werden [10, 11]. Denn es gilt: Je früher Kinder und Jugendliche mit Alkohol in Kontakt kommen, desto größer ist das Risiko einer späteren Abhängigkeit im Erwachsenenalter [12]. Präventionseffekte stellen sich insbesondere dann ein, wenn Kinder und Jugendliche früh erreicht und in Maßnahmen eingebunden werden.

## Die Kommune als zentrales Setting der Sucht- bzw. Alkoholprävention

In der Sucht- und Alkoholprävention gilt der Settingansatz (zur Umsetzung der Prinzipien der Ottawa-Charta, WHO 1986) als Schlüsselstrategie, die sich nach dem Modell von Kilian et al. [13] zwischen einer Verhaltensorientierung (Stärkung individueller Kompetenzen und Ressourcen) und einer Verhältnisorientierung im Sinne einer Strukturentwicklung gesundheitsförderlicher Lebens‑, Lern- und Arbeitsbedingungen aufspannt. Die Kommune wird im Settingansatz als das zentrale Setting betrachtet, das alle Lebenswelten – wie etwa Bildungseinrichtungen, Betriebe und Arbeitsstätten, Freizeiteinrichtungen – umfasst und einen unmittelbaren Zugang zu den Menschen ermöglicht. Außerdem ist die Kommune „Plattform für präventive Interventionen“ und gleichzeitig „Gegenstand der [gesundheitsförderlichen] Entwicklungen“ [14]. Um das präventive Potenzial bestmöglich entfalten zu können, sollten Strukturen, Prozesse, Angebote und Werte der Kommune gemeinsam betrachtet und entwickelt werden, was den Settingansatz zu einem teilweise normativen Konzept macht, dessen Umsetzung komplex ist [14]. Folgend werden Rahmenbedingen und Organisationsstrukturen der Alkoholprävention in Kommunen betrachtet.

### Organisationsstruktur der kommunalen Alkoholprävention

Gemäß Art. 28 Absatz 2 Grundgesetz (GG), der die kommunale Selbstverwaltung regelt, ist den Städten und Gemeinden das Recht garantiert, sämtliche Angelegenheiten der örtlichen Gemeinschaft im Rahmen der Gesetze selbst zu regeln. Entsprechende Zuständigkeiten sind in Kommunalverfassungen bzw. Gemeindeordnungen festgelegt, die auf Ländergesetzgebungen basieren. Zwar zählt die Gesundheitsförderung im Sinne des Schutzes vor gesundheitlichen Gefährdungen zu den kommunalen Pflichtaufgaben – als jüngstes Beispiel können zahlreiche Maßnahmen zur Eindämmung der Coronapandemie genannt werden –, die Suchtprävention ist hingegen keine kommunale Pflichtaufgabe.

Die realen Leistungen zur Erfüllung dieser freiwilligen Aufgabe werden überwiegend durch freie Träger erbracht, die kommunal oder durch Landeszuwendungen finanziert werden [1]. In der Regel werden dort Suchtpräventionsfachkräfte beschäftigt, die für die Umsetzung konkreter Maßnahmen vor Ort zuständig sind. Zu deren Aufgabenfeldern gehören u. a.:

- die Initiierung, Koordinierung und Durchführung von Präventionsmaßnahmen vor Ort,
- die Information und Aufklärung über Ursachen von Sucht und deren Prävention,
- die Beratung von Interessierten und Betroffenen, Multiplikatorinnen und Multiplikatoren sowie Institutionen zu Fragen der Suchtprävention,
- die Durchführung von Qualifizierungs- und Fortbildungsmaßnahmen für haupt- und ehrenamtlich Mitarbeitende.

Zur Koordinierung und Steuerung dieser Aufgaben werden häufig institutionalisierte Strukturen, wie beispielsweise Fach- und Arbeits- oder Koordinierungskreise oder Präventionsräte, geschaffen, die Sucht- und Alkoholprävention optimalerweise im Sinne einer Querschnittsaufgabe (also durch alle beteiligten Personen bzw. Institutionen) bearbeiten. Zu den kommunalen Akteuren, die in die Bearbeitung dieser Aufgabe involviert sind, zählen Gesundheits‑, Sozial- und Ordnungsämter, Fachstellen für Suchtprävention, kommunale Suchtbeauftragte, kommunale Präventionsräte, aber auch Wohlfahrtsverbände, Nichtregierungsorganisationen (NRO), Selbsthilfegruppen, lokale Initiativen oder die örtlichen Polizeibehörden etc.

Trotz unterschiedlicher Ausprägungen, Herangehensweisen und Zuständigkeiten herrscht Konsens darüber, dass „Suchtprävention am besten vor Ort beginnt“ [15]. Außerdem wird bei der praktischen Umsetzung „im Hinblick auf Betreuungs‑, Beratungs- und allgemeine Präventionsaktivitäten in einigen Bundesländern die Zuständigkeit der Kommunen in den letzten Jahren verstärkt betont“ [4]. Vorteilhaft ist in dem Kontext, dass die Kommunen „am besten die Probleme vor Ort [kennen] und die lokalen Akteure des Dritten Sektors bzw. der Zivilgesellschaft, die einen Beitrag zur Lösung leisten können“ [1].

### Strategien der kommunalen Alkoholprävention

Für die kommunale Alkoholprävention wird eine strategische Ausrichtung im Sinne einer Kombination aus verhältnis- und verhaltenspräventiven Maßnahmen als sinnvoll erachtet [13, 17]. Als Vorbild könnte die Tabakprävention dienen: In den letzten 10 Jahren hat sich gezeigt, dass gerade die Kombination verhältnispräventiver Maßnahmen (Tabakkontrolle, Nichtraucherschutzgesetz, Tabaksteuererhöhung, Werbebeschränkungen, Abgabebeschränkungen etc.) und verhaltenspräventiver Maßnahmen (Aufklärungs- und Informationskampagnen, Förderung der Tabakentwöhnung etc.) zur Erreichung der nationalen Gesundheitsziele einen beträchtlichen Beitrag leisten kann [16]. Tendenziell ist eine Orientierung an dem in der Tabakprävention erfolgreich praktizierten „Policy-Mix“ (Kombination verhältnis- und verhaltenspräventiver Maßnahmen) hilfreich. Ein bedeutender Unterschied besteht allerdings im Hinblick auf gesetzgeberische Möglichkeiten. Rauchverbote können etwa mit dem direkten Nichtraucherschutz und der Schädigung Dritter durch das Passivrauchen begründet werden. Eine Fremdschädigung durch Alkoholkonsum wird hingegen von der Rechtsprechung nicht festgestellt. Konsumbeschränkungen im öffentlichen Raum sind schwierig umzusetzen, denn das Grundgesetz Art. 2 Abs. 1 schützt die allgemeine Handlungsfreiheit [18].

Nichtsdestotrotz sind Maßnahmen der kommunalen *Verhältnisprävention* oft politisch orientiert. Sie setzen auf eine Veränderung und Beeinflussung der sozialen, kulturellen, rechtlichen und ökonomischen Rahmenbedingungen innerhalb einer Kommune. In entsprechenden Einrichtungen oder gesellschaftlichen Bereichen werden Strukturen und Regeln so gestaltet bzw. beeinflusst, dass Rauschtrinken eingeschränkt oder verhindert wird. Hierzu zählen insbesondere [18]:

- Alkoholkonsumbeschränkungen bzw. -verbote in öffentlichen Bereichen, Beschränkungen des Mitsichführens von Alkohol,
- Alkoholkonsumbeschränkungen bzw. -verbote im öffentlichen Personennahverkehr (ÖPNV) und deren Überwachung,
- Alkoholverkaufsverbote und deren Überwachung,
- Alkoholsteuererhöhungen,
- Alkoholtestkäufe und Schulungen des Verkaufspersonals,
- Verzicht auf Alkoholwerbung auf kommunalen Werbeflächen,
- Erhöhung des Kontrolldrucks/Alkoholkontrollen im Straßenverkehr,
- Bereitstellung eines attraktiven Angebots an alkoholfreien Getränken bei Festen und Veranstaltungen.

Zum Spektrum *verhaltenspräventiver Interventionen* zählen solche, die sich auf Wissen, Einstellungen und Verhalten einzelner Personen oder Gruppen beziehen und häufig mit pädagogischen Methoden versuchen, Individuen zu einem gesundheitsbewussten Verhalten zu bewegen. Hierzu gehören vor allem [17]:

- Förderung von Lebens- und Risikokompetenzen, z. B. durch erlebnispädagogische Maßnahmen,
- Peer-Education (sozialpädagogischer Arbeitsansatz, der vorsieht, dass speziell ausgebildete Jugendliche Gleichaltrige über die Risiken des Alkoholkonsums informieren),
- Alternative Freizeitangebote, die unter Einbeziehung der Zielgruppe entwickelt werden,
- Streetworking und aufsuchende Hilfe und Beratung,
- Informations- und Aufklärungsmaßnahmen zur Verhinderung von Rauschtrinken,
- Förderung von Punktnüchternheit, etwa im Straßenverkehr.

Weitere unterstützende Maßnahmen, die *sowohl verhaltens- als auch verhältnispräventiv* ausgerichtet sein können, sind etwa [17]:

- Fortbildung von Multiplikatorinnen und Multiplikatoren,
- Medienarbeit und Öffentlichkeitskampagnen,
- Erarbeitung von Leitfäden, Arbeitshilfen, Infomaterialien.

**Figure 3:**
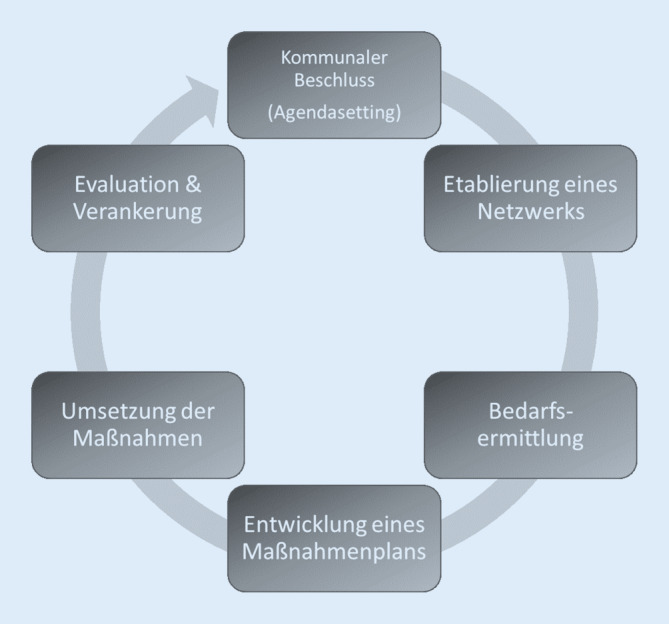

Durch den Auf- bzw. Ausbau von alkoholpräventiv agierenden Netzwerken kann Präventionsarbeit nachhaltig in allen Lebenswelten im Dachsetting Kommune verankert werden. Ein Netzwerk sollte stets unter Einbeziehung kommunalpolitischer Entscheidungstragenden agieren, da die Kommunalpolitik die maßgeblichen Rahmenbedingungen für die Umsetzung einer alkoholpräventiven Strategie setzen kann. Bestenfalls wird die Präventionsarbeit dabei durch kommunalpolitische Beschlüsse gestützt. Eine 6‑stufige Strategie zur Umsetzung von kommunalen Alkoholpräventionsvorhaben hat Jordi mit dem – auf die Alkoholprävention angepassten – Konzept des politischen Zirkels (Policy Cycle) vorgelegt (Abb. 3; [19]).

Inzwischen wurde dieses Modell in verschiedenen Projekten umgesetzt (s. unten; [19, 31]). Eine wichtige Voraussetzung für die Umsetzung alkoholpräventiver Aktivitäten ist aber immer der *politische Wille*. Dieser ist insbesondere dann ausgeprägt, wenn sich in der Kommune aufgrund eines Missstands ein Handlungsdruck aufbaut, der durch die breite und mediale Öffentlichkeit verstärkt wird [20].

### Herausforderungen der kommunalen Alkoholprävention

Folgend werden die Herausforderungen der kommunalen Alkoholprävention skizziert, mit denen Kommunen in unterschiedlichen Bereichen konfrontiert sind:

#### Folgen des Rauschtrinkens im öffentlichen Raum.

Durch das Rauschtrinken im öffentlichen Raum kommt es zum Beispiel zu nächtlichen Ruhestörungen in der Nähe von öffentlichen Plätzen oder Ausschankbetrieben, die ein behördliches Einschreiten erfordern. Wesentlich aufwendiger gestaltet sich mitunter der Umgang mit rauschtrinkenden Jugendlichen auf An- und Abreisewegen zu Veranstaltungen oder Nachtclubs. Fußballspiele oder Großveranstaltungen (Volksfeste, Musikkonzerte, Kirmes, Karneval etc.) sind ebenfalls Anlässe, um Rauschtrinken zu betreiben. In vielen Städten wird darüber hinaus von Personengruppen in Parks oder auf öffentlichen Plätzen Alkohol in großen Mengen konsumiert. Im Zusammenhang mit dem Rauschtrinken im öffentlichen Raum stehen außerdem folgende Punkte:

- Alkoholintoxikationen: Besonders gefährdet sind Jugendliche und junge Erwachsene. Mehr als 14.900 Jugendliche im Alter von 10–17 Jahren wurden im letzten Erhebungsjahr (2018) mit einer Alkoholvergiftung ins Krankenhaus eingeliefert [21].
- Gewaltkriminalität im Sinne der polizeilichen Kriminalstatistik^2^: Etwa ein Drittel aller durch die Behörden aufgeklärten Fälle von Gewaltkriminalität werden unter Alkoholeinfluss verübt [22].
- Teilnahme am Straßenverkehr unter Alkoholeinfluss: Im Jahr 2018 ereigneten sich rund 35.600 Unfälle in Deutschland, bei denen mindestens ein Beteiligter unter Alkoholeinfluss stand [23].

Zusätzlich verzeichnen deutsche Städte und Gemeinden nicht zu beziffernde Fälle von Vandalismus gegen öffentliches und privates Eigentum. Häufig stehen diese im Zusammenhang mit Rauschtrinken. Ebenso lassen sich viele Störungen der öffentlichen Sicherheit und Ordnung auf Rauschtrinken zurückführen.

#### Folgen einschränkender Maßnahmen auf ökonomischer Ebene.

Die Bekämpfung der genannten Probleme ist ihrerseits ebenfalls mit Herausforderungen verbunden, zum Beispiel im Bereich der Wirtschafts- und Finanzpolitik. Einschränkungen oder gar Verbote des Alkoholkonsums oder -verkaufs sorgen etwa für niedrigere Gewerbesteuereinnahmen. Regionen, in denen die Produktion von alkoholischen Getränken wie Bier oder Wein als tradiertes Kulturgut betrachtet wird, befürchten bei einer Verhängung von Alkoholkonsumverboten Nachteile in der touristischen Vermarktung. Rückgänge im Tourismus können zu direkten finanziellen Einbußen im kommunalen Haushalt führen. Gleiches gilt für die Einschränkung des Alkoholkonsums auf Festen und Veranstaltungen. Häufig werden Kosten für kulturelle Programme durch Einnahmen aus dem Alkoholverkauf (mit-)finanziert. Außerdem wertet das Angebot jugendaffiner alkoholischer Getränke das angestaubte Image manches Dorffestes auf, verführt gleichzeitig aber das jüngere Publikum zum Rauschtrinken [15]. Dies bringt Kommunalpolitikerinnen und -politiker in einen Interessenskonflikt zwischen der Förderung von Präventionshandeln und der attraktiven Vermarktung der Kommune.

#### Intra- und interpersonelle Konflikte bei Politiker/innen.

Siebenhüter diagnostiziert des Weiteren eine hohe Responsivität zwischen Bürgerinnen und Bürgern (Wählerinnen und Wählern) und Kommunalpolitikerinnen und -politikern (Gewählten). Politikerinnen und Politiker müssen, wenn sie die kommunale Alkoholprävention vorantreiben wollen, am Gemeinwohl orientierte Maßnahmen treffen, die aber als unpopulär oder freiheitseinschränkend empfunden werden können. Gleichzeitig nehmen Kommunalpolitikerinnen und -politiker mit ihren Wählerinnen und Wählern gemeinsam am kommunalen Leben teil. Oft sind sie in denselben Brauchtumsvereinen engagiert, in denen entgegengesetzte Interessen vertreten werden und der Konsum von Alkohol möglicherweise einen hohen Stellenwert genießt. Lokalpolitikerinnen und -politiker stehen somit in einem intra- und interpersonellen Konflikt in Bezug auf die Umsetzung alkoholpräventiver Maßnahmen [15].

#### Begrenzte Fördermittel.

Als eine weitere bedeutsame Herausforderung beklagen die freien Träger und Initiativen der Suchthilfe und Suchtprävention eine Unterfinanzierung des Bereichs infolge stagnierender kommunaler Finanzierungsbeiträge. Das Leibniz-Institut für Wirtschaftsforschung kommt zu der Auffassung, dass kommunale Fördermittel zwar erhöht wurden, die Träger jedoch weder mit wachsenden Aufgaben noch mit wachsenden Kostensteigerungen Schritt halten können [1].

#### Wirksamkeit der Präventionsprojekte.

Als letzten Aspekt sind kommunale Alkoholpräventionsprojekte kritisch im Hinblick auf ihre Wirksamkeit zu betrachten. Vielerorts besteht nicht die Möglichkeit, nachzuweisen, wie effektiv eigeninitiierte Interventionen sind. Selbst wenn Budgetierungen für Evaluationen vorhanden sind, sind diese meist knapp bemessen und analysieren daher häufig eher den Prozess (Prozessevaluation) – also *wie *eine Intervention funktioniert. Seltener wird eine Ergebnisevaluation ermöglicht, durch die belegt werden kann, dass eine Intervention die gewünschte Wirkung erzielt [24]. Eine Übersichtsarbeit zur Wirksamkeit kommunaler Alkoholpräventionsprojekte fehlt aktuell noch [25]. Dies ist angesichts der „außerordentliche(n) Komplexität kommunaler Prävention“ auch nicht verwunderlich, allerdings dennoch nötig. Um Gesamteffekte von Maßnahmen in mehreren Kommunen miteinander vergleichen zu können, bedarf es allerdings aufwendiger Evaluationsdesigns. Aufgrund von geringer Standardisierung und unterschiedlichen Rahmenbedingungen für kommunale Interventionen könnten am Ende möglicherweise wenig aussagekräftige Ergebnisse stehen [25]. Weiterhin gilt: Eine „kommunale Alkoholpolitik, die unter Einbeziehung lokaler Stakeholder mehrere Regulierungen umsetzt (Beschränkung des Zugangs zu Alkohol, seine Verfügbarkeit, Alkohol im Straßenverkehr), könnte präventive Effekte auf die negativen Folgen des Alkoholkonsums haben“ [25].^3^

## Modellprojekte und -programme der kommunalen Alkoholprävention

Entgegen der oben beschriebenen „Evidenzarmut“ sind in den vergangenen Jahren einige Modellprojekte mit unterschiedlichen Ansätzen für kommunale Alkoholprävention erprobt, evaluiert und zum Teil verstetigt worden. In diesem Abschnitt werden 4 dieser Projekte exemplarisch vorgestellt:

### Hart am Limit (HaLT)

„HaLT – Hart am Limit“ ist ein Programm zur kommunalen Alkoholprävention. Das ursprünglich als selektiv bzw. indiziert^4^ konzipierte Präventionsprojekt richtet sich an Kinder und Jugendliche, die durch Rauschtrinken auffällig wurden. Wesentlicher Bestandteil ist das sogenannte Brückengespräch, bei dem Jugendliche nach einer Alkoholintoxikation noch im Krankenhaus zur Reflexion ihres Alkoholkonsums aufgefordert werden. Inzwischen ist dieser reaktive Programmbestandteil durch einen proaktiven Baustein, der alkoholpräventive Maßnahmen in Kommunen fördert, erweitert worden (z. B. die Qualifizierung von Fachkräften und die Unterstützung von Koordination und Vernetzung). Rund 160 Standorte in 14 Bundesländern nehmen aktuell an dem Programm teil. Eine Ergebnisevaluation mit positiven Wirksamkeitsnachweis wurde durchgeführt [26]. (Für weitere Informationen siehe auch Beitrag von Eichin et al. in diesem Themenheft.)

### Bundeswettbewerb „Vorbildliche Strategien kommunaler Suchtprävention“

Seit 2001 wird regelmäßig der Bundeswettbewerb „Vorbildliche Strategien kommunaler Suchtprävention“ von der BZgA gemeinsam mit der Drogenbeauftragten der Bundesregierung und mit Unterstützung der kommunalen Spitzenverbände sowie des GKV-Spitzenverbandes ausgeschrieben. Der Wettbewerb hat wechselnde Themenschwerpunkte. Ziel des Wettbewerbs ist es, vorbildliche Strategien der kommunalen Suchtprävention zu identifizieren und bekannt zu machen, sodass sie für andere Kommunen eine Inspiration sein können [27].

### STAD in Europe

Das Projekt STAD in Europe (SiE) ist ein im Jahr 2016 initiiertes Modellprojekt nach schwedischem Vorbild (STAD: Stockholm prevents alcohol and drug problems). Es wird EU-weit in 7 Ländern durchgeführt und von der Europäischen Kommission gefördert. Ziel des Projekts ist es, mehr Wissen über effektive Strategien zur Verringerung von Alkoholexzessen zu verbreiten. Dies soll vor allem über die Einschränkung der Verfügbarkeit von Alkohol im Setting Nachtleben und anderen „Trinkumwelten“ erreicht werden. Als Evaluationsergebnis liegen inzwischen ein systematischer Präventionsansatz, ein evidenzbasiertes Strukturmodell sowie eine wissenschaftlich fundierte Interventionsstrategie vor [28].

### GigA (Gemeinsam initiativ gegen Alkoholmissbrauch bei Kindern und Jugendlichen) 2010–2020

Das Projekt GigA wurde im Rahmen der BZgA-Kampagne „Alkohol? Kenn dein Limit.“ umgesetzt und evaluiert. Mithilfe des Netzwerkbezogenen Qualitätsmanagements (NBQM) des Landschaftsverbands Rheinland (LVR) wurde in Kommunen ein abgestimmtes Zusammenwirken aller an Alkoholprävention beteiligten Institutionen und Professionen sichergestellt. Vorhandene regionale Konzepte und Handlungsstrategien der beteiligten Akteure wurden zu einer gemeinsamen Arbeitsgrundlage koordiniert und vernetzt mit dem Ziel, gemeinsame Maßnahmen zur Prävention des Rauschtrinkens bei Kindern und Jugendlichen umzusetzen. Im Laufe des Projekts konnten so in über 40 Kommunen alkoholpräventive Netzwerke etabliert werden [29]. Die Ergebnisse des Projekts GigA flossen im Jahr 2020 in die Entwicklung der neuen BZgA-Serviceplattform für kommunale Alkoholprävention VORTIV – vor Ort aktiv www.vortiv.de ein (siehe unten).

Betrachtet man die Ziele und Ausrichtungen der oben aufgeführten Projekte, so wird rasch deutlich, in welchen Bereichen kommunale Alkoholprävention Unterstützung erfahren sollte: in der Schaffung einer funktionstüchtigen Strukturentwicklung, der Bildung von ressort-/institutionsübergreifenden Netzwerken und der Verankerung einer nachhaltigen Alkoholprävention. Ebendiese Punkte werden in den genannten Modellprojekten aufgegriffen und in die Kommunalstrukturen eingegeben. So sollen das Präventionshandeln vor Ort und die ausführenden Träger und Suchtpräventionsfachkräfte gestärkt werden.

Um die Stärkung der kommunalen Alkoholprävention noch zielgerichteter und passgenauer voranzutreiben, startete 2020 die BZgA-Serviceplattform „VORTIV – vor Ort aktiv“ [30]. VORTIV richtet sich an alle kommunalen Akteure. Bedarfe in Kommunen werden präzise analysiert und Akteure bei der Entwicklung von Präventionsstrategien und bei der Planung, Umsetzung und Evaluation von kommunalen Maßnahmen individuell beraten. Darüber hinaus wird ein Fortbildungsmodul zum qualitätsgesicherten kommunalen Netzwerkmanagement angeboten und es werden Suchtpräventionsfachkräfte bei der Initiierung bzw. Reaktivierung ihrer Netzwerke beraten, begleitet und unterstützt. Sämtliche BZgA-Angebote zur Alkoholprävention können über www.vortiv.de abgerufen und vor Ort umgesetzt werden. Eine stetige Weiterentwicklung in einem partizipativen Prozess und die Einrichtung digitaler Zusammenarbeits- und Austauschmöglichkeiten sind vorgesehen.

## Fazit

Kommunen sind die zentrale Lebenswelt der Bevölkerung und deshalb von wesentlicher Bedeutung bei der Umsetzung alkoholpräventiver Maßnahmen. Die kommunale Alkoholprävention ist aber gleichzeitig eine von vielen Herausforderungen geprägte Aufgabe, denn Alkohol ist als Kulturgut tief verwurzelt, was sich auch in den kommunalen Rahmenbedingungen widerspiegelt.

Um alkoholpräventive Interventionen in Kommunen konsequent voranzutreiben, bedarf es eines gut funktionierenden Netzwerks von Akteuren. Dieses Netzwerk sollte systematisch und zielorientiert wirksame Maßnahmen planen, umsetzen und evaluieren. Ein solches Netzwerk umfasst optimalerweise alle Akteure, die in der Kommune mit dem Thema Alkoholprävention betraut sind. Kommunale Netzwerke dieser Art kann es auf Dauer nur geben, wenn die kommunalen Entscheidungstragenden die Alkoholprävention in ihrer politischen Agenda nach oben stellen. Noch besser ist es, wenn kommunale Entscheidungstragende im Falle von größerem Handlungsbedarf auch die Verabschiedung regulatorischer Bestimmungen nicht scheuen und sich dafür einsetzen.

Aktuell fehlen noch vollständige Übersichten über wirksame kommunale Alkoholpräventionsstrategien oder -projekte. Dies begründet sich in der Komplexität dieses Tätigkeitsbereichs. Ein entsprechendes Evaluationsdesign, um die Wirkung von kommunalen Alkoholpräventionsstrategien zu belegen, ist in der Konzeption aufwendig und in der Durchführung kostenintensiv. Nichtsdestotrotz sollte die Präventionsforschung auch diesen Bereich mithilfe komplexitätsreduzierender Untersuchungsdesigns zukünftig besser ausleuchten, damit die relevanten Parameter erfolgreicher kommunaler Alkoholprävention identifiziert werden können. Dies würde perspektivisch eine Optimierung der Evidenzbasierung in der kommunalen Alkoholprävention fördern.

Festzuhalten bleibt: Gelingende Alkoholprävention in Kommunen ist eine vielschichtige und komplexe Aufgabe. Sie gelingt vor allem dann, wenn kommunale Entscheidungstragende sie unterstützen und die Bildung von Netzwerken ermöglichen, die sich dem Thema als Querschnittsaufgabe widmen. Diese Netzwerke sollten auf Dauer angelegt sein und sich bei der Planung, Umsetzung und Evaluation ihrer kommunalen Strategien und Maßnahmen an evidenzbasierten Angeboten orientieren und so vom Know-how-Transfer profitieren.

## Footnotes

1 Rauschtrinken bezeichnet den Konsum von 5 (männliches Geschlecht) bzw. 4 (weibliches Geschlecht) Standardgläsern mit 10–12 g Reinalkohol bei einer Trinkgelegenheit.

2 Zur Gewaltkriminalität werden in der polizeilichen Kriminalstatistik aktuell folgende Delikte gezählt: Mord, Totschlag, Tötung auf Verlangen, Vergewaltigung, sexuelle Nötigung, sexueller Übergriff im besonders schweren Fall einschließlich mit Todesfolge, Raub, räuberische Erpressung, räuberischer Angriff auf Kraftfahrer, Körperverletzung mit Todesfolge, gefährliche und schwere Körperverletzung, Verstümmelung weiblicher Genitalien, erpresserischer Menschenraub, Geiselnahme und Angriff auf den Luft- und Seeverkehr. Weil sich einzelne Gesetze im Laufe der Jahre verändern, kann es immer wieder zu neuen Zuordnungen kommen.

3 Im Memorandum Evidenzbasierung in der Suchtprävention [31] werden Handlungsempfehlungen zu einer besseren Verzahnung in der Kooperation zwischen Forschung und Praxis im Hinblick auf die Evidenzbasierung bzw. Evidenzgenerierung suchtpräventiver Maßnahmen gegeben. Grundlegend wird der Suchtpräventionsforschung die Aufgabe attestiert, „bestmöglich wissenschaftliches Wissen zu generieren, das den Kriterien für solches genügt und das für evidenzbasiertes Handeln nutzbar gemacht werden kann und soll“ [31]. Dieses Wissen sollte stets für Praxisakteure handhabbar gemacht werden, wie z. B. die 12 Good-Practice-Kriterien des Kooperationsverbunds Gesundheitliche Chancengleichheit in der Gesundheitsprävention [32].

4 Selektive bzw. indizierte Präventionsprojekte richten sich an Personen, bei denen eine Problemindikation bereits besteht. In diesem Fall an Jugendliche, die durch Rauschtrinken auffällig und mit einer Alkoholintoxikation ins Krankenhaus eingeliefert wurden. Im Gegensatz dazu richten sich universelle Präventionsprojekte an alle Personen der jeweiligen Bevölkerungsgruppe.
